# Emerging role of circulating tumor cells in immunotherapy

**DOI:** 10.7150/thno.59677

**Published:** 2021-07-06

**Authors:** Alexey Rzhevskiy, Alina Kapitannikova, Polina Malinina, Arthur Volovetsky, Hamidreza Aboulkheyr Es, Arutha Kulasinghe, Jean Paul Thiery, Anna Maslennikova, Andrei V. Zvyagin, Majid Ebrahimi Warkiani

**Affiliations:** 1 ARC Centre of Excellence for Nanoscale BioPhotonics, Macquarie University, Sydney, NSW 2109, Australia; 2 Institute of Molecular Medicine, Sechenov First Moscow State Medical University, 119991 Moscow, Russia; 3 Institute for Urology and Reproductive Health, Sechenov University, Moscow 119991, Russia; 4 Privolzhsky Research Medical University, 10/1, Minini Pozharsky Square, Nizhny Novgorod 603005, Russia; 5 Lobachevsky State University of Nizhny Novgorod, Gagarina Avenue 23, Nizhny Novgorod 603950, Russia; 6 School of Biomedical Engineering, University of Technology Sydney, 2007 Sydney, Australia; 7 Queensland University of Technology, Centre for Genomics and Personalised Health, School of Biomedical Sciences, Faculty of Health, Woolloongabba, QLD 4102, Australia; 8 Translational Research Institute, Woolloongabba, QLD 4102 Australia; 9 Guangzhou Institutes of Biomedicine and Health, Guangzhou, People's Republic of China; 10 The Chair of Cancer, Radiotherapy and Radiologic Diagnostics, Privolzhsky Research Medical University, Nizhniy Novgorod. Russia 603005; 11 IBCh - Shemyakin Ovchinnikov Institute of BioOrganic Chemistry of the Russian Academy of Sciences, Miklukho Maklai Street, 16, Moscow, Russia

**Keywords:** Immunotherapy, circulating tumor cells, immune checkpoint inhibitors, cancer, personalized medicine

## Abstract

Over the last few years, immunotherapy, in particular, immune checkpoint inhibitor therapy, has revolutionized the treatment of several types of cancer. At the same time, the uptake in clinical oncology has been slow owing to the high cost of treatment, associated toxicity profiles and variability of the response to treatment between patients. In response, personalized approaches based on predictive biomarkers have emerged as new tools for patient stratification to achieve effective immunotherapy. Recently, the enumeration and molecular analysis of circulating tumor cells (CTCs) have been highlighted as prognostic biomarkers for the management of cancer patients during chemotherapy and for targeted therapy in a personalized manner. The expression of immune checkpoints on CTCs has been reported in a number of solid tumor types and has provided new insight into cancer immunotherapy management. In this review, we discuss recent advances in the identification of immune checkpoints using CTCs and shed light on the potential applications of CTCs towards the identification of predictive biomarkers for immunotherapy.

## Introduction

**Figure 1:**
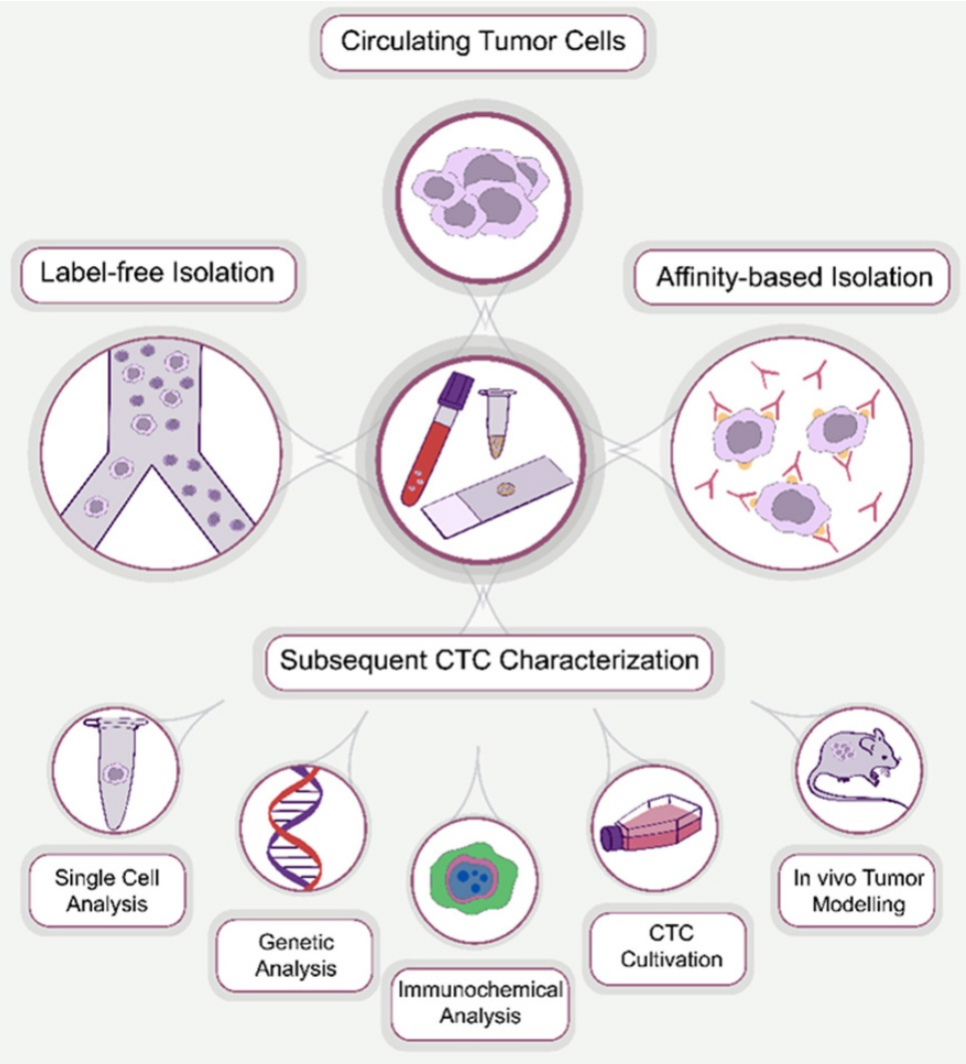
CTC processing methods. Generally, the approaches used for the enrichment of CTCs can be divided into two main groups: affinity-based and affinity-independent (label-free). After enrichment, CTCs are subjected to subsequent characterization, including single cell, genetic and immunocytochemical analysis, cultivation and in vivo tumor modeling.

Circulating tumor cells (CTCs) were first discovered in 1869 by an Australian physician, Thomas Ashworth, who identified cells that were morphologically similar to those of the primary tumor in the blood of metastatic cancer patients 1. CTCs were defined as single cells or cell clusters that are metastatic precursor cells responsible for the development of metastasis 2. They have gained significant attention as a promising biomarker for the diagnosis and prognosis of malignant tumors. However, at the early stages of cancer, CTCs are rare, typically several CTCs per milliliter of peripheral blood on an excessively high background of normal blood cells 3. This necessitates enrichment of CTCs to apply analytical protocols. Numerous methods have been introduced for the enrichment of CTCs, which are classified based on their operational principle into affinity-based and label-free isolation approaches (Figure 1).

The practical value of CTCs for clinical applications has been considered limited owing to inefficient CTC capture methodologies 4. The FDA-approved CellSearch (Menarini Silicon Biosystems) technology has been applied for CTC enumeration across a number of tumor types 5. In the CellSearch studies, EpCAM-positive CTCs counts were associated with progression-free survival (PFS) and overall survival (OS) in metastatic prostate, colorectal and breast cancer. However, later, it was identified that this approach had a relatively low efficiency of identifying CTCs at the early stages of various cancer types 6. On the other hand, the rapid development of microelectromechanical systems over the last 20 years has considerably advanced the CTC enrichment technologies, which has resulted in an increase of CTC's yield available for analysis and processing. As a result, several viable approaches for epitope dependent/independent CTC enumeration and detection have emerged. The extensive development of CTC enrichment technologies has reached a point at which the main challenge has shifted towards molecular and genetic analysis of CTCs captured by different technologies, identification of CTC subpopulations and correlative analysis of the total CTC enumeration and subpopulations.

In turn, immunotherapy has revolutionized the field of oncology by providing a mechanism for the activation of immune cells that can recognize and destroy cancer cells 7. This is realized by identifying and applying immune checkpoint inhibitors (ICIs) that target immune checkpoint proteins expressed by immune cells and cancer cells. This leads to downregulation of immune cell activation and surveillance 8, which leads to suppressed T-cell activity. The described mechanism is mediated through the binding of cytotoxic T-lymphocyte-associated antigen 4 (CTLA-4) carried by T-cells to inhibitory proteins CD80/CD86 located on the surface of antigen-presenting cells, which are predominantly situated in lymph nodes. Alternatively, T-cell suppression can be achieved by binding a programmed death 1 (PD-1) protein, also expressed on the surface of T-cells, to its ligand (PD-L1) on the surface of either T-cells or tumor cells predominantly located in the tumor microenvironment 9. Numerous drugs that block either CTLA-4 or PD-L1/PD-1 interactions have been developed and tested in various clinical trials, including ipilimumab and tremelimumab for CTLA-4, nivolumab, pembrolizumab and cemiplimab for PD-1, and atezolizumab, durvalumab and avelumab for PD-L1. Thus, assessment of the status of immune checkpoints in patients is thought to be one of the crucial aspects in personalized immunotherapy 10, 11. Recent studies have highlighted that CTCs frequently express PD-L1, which could provide a useful and non-invasive means to assess PD-L1 status in real-time 12, 13.

In this review, we discuss the emerging role of CTCs as a biomarker in evaluating the efficiency of immunotherapy in various types of solid tumors, particularly with ICIs either alone or in combination with other immunotherapy approaches. Additionally, the diagnostic and prognostic potential and the role of CTCs in personalized cancer treatment is discussed. The review is focused on urologic, gynecologic, breast, lung, head and neck, gastrointestinal cancers and melanoma.

## Search strategy

PubMed has been chosen as the data base of relevant publications. For the current review, full-text articles in English published in peer reviewed journals were selected. Also, ClinicalTrials.gov was searched for relevant clinical trials in which cancer immunotherapy was performed along with the qualitative or quantitative assessment of CTCs. The databases were searched up to December 2020. The keywords used in the literature search were as follows: circulating tumor cells, CTC(s), immunotherapy, immune checkpoint inhibitor(s), immune checkpoint blockade, immune checkpoint(s), programmed cell death protein 1, PD-1, cytotoxic T-lymphocyte-associated protein 4, CTLA-4, programmed death-ligand 1, PD-L1, bladder cancer, urothelial carcinoma, transitional cell carcinoma, prostate cancer, kidney cancer, renal cancer, renal cell carcinoma, ovarian cancer, endometrial cancer, cervical cancer, breast cancer, head and neck cancer, head and neck squamous cell carcinoma, oropharyngeal cancer, hypopharyngeal cancer, laryngeal cancer, lip cancer, oral cavity cancer, nasopharyngeal cancer, paranasal sinus cancer, nasal cavity cancer, salivary gland cancer, thyroid cancer, esophageal cancer, gastric or stomach cancer, colorectal cancer, pancreatic cancer, liver cancer, melanoma.

## Urologic cancers

Immunotherapy of the non-muscle-invasive form of bladder cancer through instillation of the bladder with Bacillus Calmette-Guerin (BCG) vaccine has been a standard of care for urologic cancers worldwide for decades 14. Recently, along with FDA approval of ICIs for the treatment of urologic cancers, promising results have been reported in studies addressing the ICI treatment of renal cell carcinoma 15, urothelial 16 cancer and the use of therapeutic vaccines for the treatment of prostate cancer (PCa) 17. Tumor mutation burden (TMB) and microsatellite instability (MSI) have been considered as promising biomarkers for the prediction of immunotherapy outcomes in urologic cancers 18. CTCs have also been actively investigated as predictive biomarkers of an immunotherapy response by measuring the expression of immune checkpoints on CTCs including PD-L1.

In urologic cancers, CTCs have been mostly investigated as predictive biomarkers in the PCa immunotherapy regimens, in which the immune checkpoint blockade (ICB) has attracted significant attention. However, monotherapy with ICB has demonstrated modest efficiency 19. At the same time, it was suggested that castration-resistant PCa expressing androgen receptor splice variant 7 (AR-V7) might be associated with a higher level of MSI and subsequently higher TMB 20. AR-V7 is an isoform of the androgen receptor 21 and can be a cause of an aggressive form of the disease and resistance to hormonal therapies, including those with enzalutamide and abiraterone 22. This calls for the development of efficient therapies of AR-V7 positive PCa to cover the unmet need, in which case ICB immunotherapy is considered a promising treatment modality.

Further, combined ICI therapies, particularly with anti-cancer vaccines 23, are of significant interest. In a preclinical study, Fu *et al.* 24 identified an immunotherapeutic mechanism of the anti-cancer vaccines that might induce an antigen-specific IFNγ-secretion by T cells in the tumor microenvironment. This leads to an increment of PD-L1 expression, with a subsequent uprise in PD-1 antibody blockade, as demonstrated by the administration of the IFNγ-inducing cancer vaccine and nivolumab in combined therapy. Therefore, such a combined approach may be beneficial in PCa immunotherapy.

In a recent study by Rekoske *et al.* 25, alterations in PD-L1 expression on CTCs of PCa patients at various stages of the disease mediated by vaccination with DNA vaccine encoding prostatic acid phosphatase were associated with elevation of the immune response and prolonged PFS. A significant increase of PD-L1 expression levels by CTCs was detected three months post-treatment, with comparable expression levels at the pre-treatment and one year post-treatment. A perspective approach of combination immunotherapy using anti-tumor vaccination and PD-1 blockade by pembrolizumab, coupled with CTCs as predictive biomarkers of PD-L1 expression, is currently being assessed in an ongoing clinical trial (NCT02499835).

A phase-2 clinical trial was conducted to evaluate the efficiency of combined ICB treatment of AR-V7 positive metastatic PCa with ipilimumab and nivolumab 26. In the study, CTCs were assessed for markers such as ARV-7, DNA-repair gene mutations and phenotypic heterogeneity, and interrelation between the markers and an outcome of the combined ICB. The results showed that the presence of various mutations in DNA-repair-related genes in isolated CTCs and their phenotypic heterogeneity were associated with improved clinical outcomes in ARV-7 positive patients.

Furthermore, the prognostic significance of PD-L1 expression in CTCs has been recently reported. In a study by Satelly *et al.*^27^, 30 metastatic PCa patients were recruited at different cycles of palliative chemotherapy and their blood was assessed for CTCs at random time points. The results showed that nuclear PD-L1 (nPD-L1) expression was associated with significantly worse PFS than OS. Furthermore, Yin *et al.* 28 reported no association between PCa stages and the expression of the antigen on CTCs, in which the expression level of PD-L1 was investigated in patients at different stages of cancer.

Urothelial bladder cancers were also identified to express PD-L1 29. In a recent study by Anantharaman *et al.* 30, CTCs were measured in 25 patients with metastatic (21 patients) and muscle-invasive (4 patients) forms of bladder cancer. Among them, CTCs were detected in blood samples of 20 patients, of which seven patients diagnosed with a metastatic form of bladder cancer showed PD-L1 expression on their CTCs. Further, four patients had more than one PD-L1 positive (PD-L1^+^) CTC per milliliter of blood, which was associated with a median OS of 194 days compared with 303 days in patients having 0-1 PD-L1^+^ CTCs per mL of blood. Noticeably, four patients had PD-L1 expression only on cytokeratin-negative cells, which may potentially provide a means for cancer cells to evade the immune system 31. Moreover, seven patients received PD-1 checkpoint immunotherapy, and data regarding the therapy outcome was available for 5 patients.

## Gynecologic cancers

Ovarian cancer (OC) is one of the most common gynecologic malignancies in women, taking third place in occurrence worldwide after cervical cancer (CC) and endometrial cancer (EC), and is ranked first in mortality in developed countries 32. In 70-80% of cases, the disease is diagnosed at its late stages, which leads to a 40-47% 5-year survival rate 33. Among all histological types of OC, 85-90% of cases have been epithelial carcinomas 34. EC is the second most common gynecologic cancer 32. Morphologically, it is divided into two major subgroups: Type I includes the most common type—endometrioid cancer (60-70% cases); Type II includes high-grade endometrioid cancer and rarer histological subtypes. Even though EC is diagnosed at an early stage, in most cases, up to 20% of tumors progress to the late stages, with a 15% 5-year survival rate 35. CC is the fourth most common cancer in women, ranking after breast cancer (2·1 million cases), colorectal cancer (0·8 million) and lung cancer (0·7 million). The 5-year survival rate for women with CC is 66%.

The “gold standard” in gynecologic cancers therapy, which has had a significant impact on improving survival rates, is the combination of surgery, radiation and chemotherapy using drugs based on taxanes and platinum 36. In the field of ovarian cancer therapy, chemotherapy with cisplatin is one of the most reliable therapies with a measurable clinical response, which is particularly effective in patients with early encapsulated epithelial or serous carcinoma 37. In endometrial cancer, combination therapy with several drugs, and the best response rate at 57% with a median survival of 15.7 months, has been achieved by therapy with carboplatin and paclitaxel with the addition of doxorubicin 38. At the initial stage of therapy, the tumor shows a high sensitivity to drugs. However, relapse usually occurs in approximately 80% of patients with ovarian cancer treated with paclitaxel or cisplatin, and in 45% of patients with endometrial cancer, also treated with cisplatin and paclitaxel 39, 40. Also, some patients develop drug resistance. Several mechanisms to explain the resistance development have been proposed, including changes in the drug absorption mechanisms by tumor cells, apoptosis inhibition and increased DNA repair processes 41. One of the reasons why drug resistance develops is associated with the role of some miRNAs' and has attracted the attention of many researchers, prompting them to look for alternative ways of tumor treatment 42. As an alternative, ICB and adoptive cell therapy represent the most actively investigated types of immunotherapy for both ovarian and endometrium cancers and antitumor vaccines for ovarian cancer 43.

Recent data have revealed that various cancers with mismatched repair deficiency are susceptible to anti-PD-1/PD-L1 immunotherapy 44. The role of PD-L1 expression on tumor cells, tissue or antigen-presenting cells in ovarian cancer is not yet well-understood 45-49. Several studies have reported a favorable prognosis for patients with PD-L1 expression on tumor cells and outcomes 47, 49, 50, whereas other studies have reported a negative impact 45, 46. Further investigation on the prognostic value of PD-L1 in ovarian cancer is required. The conclusions of clinical studies on PD-1/PD-L1 immunotherapy in ovarian cancers are very contradictory. Despite the immunotherapy efficiency was proven in many types of cancer, no immunotherapy has been approved for clinical applications in gynecologicl cancers to date. Platinum-resistant ovarian cancer is often referred to as a “cold tumor” due to decreased tumor infiltration by immune cells. Although ovarian cancer has been proven to be immune-sensitive, monotherapy with ICIs showed low efficacy (KEYNOTE-100 trial 51, JAVELIN 200 trial 52). When treatment with PD-1/PD-L1 inhibitors was combined with other therapeutic agents, an efficacy remained relatively low (phase II randomized trial NRG-003 53).

For endometrial cancer, the use of immuno-oncological drugs is justified in first line as a mono-therapy for MSI tumors (KEYNOTE-158 54), as well as in 2 and subsequent lines after progression with platinum drugs in combination with tyrosine kinase inhibitors (KEYNOTE-775). Pembrolizumab monotherapy demonstrated durable antitumor activity and manageable safety in PD-L1^+^ patients with advanced CC (KEYNOTE-158 54), which can also be taken into account and used, among other aspects, to identify PD-L1 CTCs in this group of patients.

The study of CTCs as a diagnostic and prognostic biomarker of immunotherapy is the most prevalent in OC and EC. Depending on the type of OC, CTCs occur in 67-99% of patients and are sub-divided into several phenotypes classified by their invasiveness 55-58. Over the last 10-12 years, the diagnostic and prognostic value of CTCs for assessing the dynamics of tumor development and monitoring the tumor response to the therapy in OC has been demonstrated in numerous studies 59-61. For example, Buderath *et al.* 62 investigated CTCs as a potential biomarker for carboplatin therapy efficacy. In this study, CTCs were isolated from the peripheral blood and analyzed by RT-PCR to detect and evaluate the expression level of EpCAM, MUC-1, CA-125 and ERCC1, which was compared with the expression level of PD-L1/PD-L2 in serum to identify possible correlations. The following statistically significant relationship was found between PD-L2 (programmed cell death 1 ligand 2) and CTCs: the presence of CTCs before the therapy was associated with a lower level of PD-L2, whereas an absence of CTCs was associated with an increased level of PD-L2. There was a moderate correlation between the decrease in PD-L2 level and resistance to platinum chemotherapy. According to recent data, CTCs were detected in 33-75% of patients and occurred if the tumor invaded the cervix 63, 64. Several EC biomarkers were identified through molecular profiling of isolated CTCs. Some of them, such as CTNNB1, GDF15, RUNX1, BRAF and PIK3CA, are believed to be worthwhile for further investigation as therapeutic targets. Moreover, the absence of CTCs was demonstrated after one cycle of standard chemotherapy in a study by Ni *et al.* 65. No associations were found between the number of CTCs and serum level of CA-125/HE4 or the expression of the other tumor biomarkers. The diagnostic and prognostic value of CTC in EC remains unclear, including monitoring the effects of various types of immunotherapies 63.

Several clinical trials of immunotherapeutic drugs have been performed to evaluate clinical applications of CTCs as a potential biomarker in treatment efficacy measurement. Berzovsky *et al.* have reported a clinical trial of a human epidermal growth factor receptor 2 (HER2)-targeted cancer vaccine 66. It was based on autologous dendritic cells taken from patients and transfected with an adenovirus expressing non-signaling extracellular and transmembrane domains of HER2. Phase I of this study involved patients with the metastatic form of ovarian adenocarcinoma who failed to respond to at least one of the standard treatment regimens and had not previously received trastuzumab or other anti-HER2 therapy. In this study, a decrease in the number of CTCs by 40%, 83% and 100% at 12, 28 and 48 weeks was demonstrated.

In addition, clinical trials of GL-ONC1 oncolytic immunotherapy, i.e., genetically modified vaccinia oncolytic viruses (VOV), have been conducted (NCT02759588) 67 to retrospectively identify the clinical benefits of VOV monotherapy in patients with platinum-resistant OC. The comparative analysis included 11 patients and the following assaying: measurement of immune competence with neutralizing antibody titers, activity of virus-encoded glucuronidase, tumor response according to RECIST 1.1, prognostic nutrition index, and CTCs. As a result, CTCs were found as a prognostically significant marker: The absence of CTCs in peripheral blood directly correlated with the positive therapeutic effects of GL-ONC1. Generally, CTCs may be considered potentially significant biomarkers in the diagnosis and prognosis of OC. However, interrelations between CTCs and different immunotherapy types remain unclarified owing to the lack of data.

## Breast cancer

Although breast cancer (BC) is not traditionally considered immunogenic 68, increasing evidence suggests that its specific molecular subtypes, namely triple-negative and HER2-positive subtypes, often correlate with substantial infiltration of immune cells with a particular prognostic and even predictive value 73. This has contributed to the ongoing assessment of ICI efficiency, especially with a triple-negative molecular subtype. In the KEYNOTE-086 study 69, an increase of objective response rate (ORR) was observed in untreated PD-L1^+^ patients with metastatic triple-negative breast cancer (mTNBC) compared with patients receiving pembrolizumab in the second and subsequent lines of therapy. The use of pembrolizumab in combination with eribulin for mTNBC (KEYNOTE-150 / ENHANCE 1 trials 70) in the first line of therapy has shown better results in PD-L1^+^ patients, as well as the use of atezolizumab in combination with nab-paclitaxel (Impassion-130 71) as well as the use of pembrolizumab in combination with nab-paclitaxel (KEYNOTE - 355 72). Based on KEYNOTE-119 73 trial, patients with overexpression of PD-L1 have also shown a good response to mono-immunotherapy with pembrolizumab in second and subsequent lines of therapy. The clinical efficacy of ICIs for the treatment of patients with HER2+ breast cancer remains to be determined. For instance, in the KATE-2 trial 74, the addition of atezolizumab to trastuzumab emtansine did not show a clinically meaningful improvement in progression-free survival and was associated with more adverse events. Thus, untreated patients with locally advanced and metastatic TNBC expressing PD-L1 may benefit most from immunotherapy in combination with chemotherapy. However, it is worth mentioning that the clinical significance of PD-L1 as a biomarker for breast cancer has not been conclusively determined 75.

An implementation of PD-L1 as a reliable biomarker for selecting or excluding patients with BC for immunotherapy was complicated by several challenges, mainly related to molecular and cellular heterogeneity of the disease and the methodology used to measure PD-L1 expression. The assessment of PD-L1 expression in BC by immunohistochemical (IHC) methods has been widely studied. PD-L1 expression was significantly higher in invasive diseases than in normal breast tissue 82 and cancer *in situ* 76. Additionally, a significant difference in PD-L1 expression was observed in different molecular subtype cohorts of BC favoring TNBC (up to 60% of PD-expression L1) 86. These data appear to be consistent with the observation that the tumor expression of PD-L1 directly correlates with the number of stromal tumor-infiltrating lymphocytes (TILs) in this breast cancer subtype 87, which is known to be more often associated with TILs infiltration.

Evaluation of the PD-L1 expression by IHC methods is challenging because it is highly variable in tumor cells. For example, HER2-positivity was associated with a higher expression of PD-L1 (up to 50%) compared with HER2-negative BC in some studies 77, 78, whereas negligible differences have been reported in other studies 90. The results of two meta-analyses studies confirmed a greater expression of PD-L1 in TNBC 92 compared with non-TNBC subtypes but were not consistent in reporting a relationship between HER2 status and PD-L1 expression. The PD-L1 expression was associated with unfavorable clinical and prognostic characteristics, such as a lower degree of differentiation 94, high proliferative index 79, advanced stage 80, larger tumor size 81 and younger age 82. However, conflicting data have been obtained regarding the association of PD-L1 expression with a better or worse clinical outcome. Some authors have reported lower disease-free survival and/or OS in cases of higher expression of PD-L1 by primary BC 83, especially in a TNBC subtype 84. In contrast, other authors have reported the expression of PD-L1 was positively and independently associated with disease-free survival and/or OS in several primary BC cohorts 85-87.

The stromal compartment appears to make a significant contribution to the expression of PD-L1 in BC 88, 89. However, no consistent data have been reported on the possible biological and clinical consequences of the differential expression of PD-L1 by CTCs or TILs. It has been suggested that evaluation of PD-L1 on stromal immune cells avoids false-negative results in the evaluation of PD-L1 status of breast cancer. This has the potential to increase the cohort of patients suitable for immunotherapy 89. The results of a Javelin phase Ib study using avelumab 90 showed that the prognostic value of PD-L1 was probably more significant when PD-L1 was evaluated on TILs rather than on CTCs. A preliminary translational analysis of the randomized phase III study of Impassion130 showed that the assessment of TILs provided no additional predictive information above that provided by PD-L1 status 91.

Available data on spatial and temporal heterogeneity of PD-L1 92 expression remain limited. These data have been largely obtained in analytical assaying of PD-L1 for metastases in the lymph nodes, in which the reliability of the determination of the immunity-related biomarkers was unclear and might be biased 93. Jilaveanu *et al.* have reported a weak correlation in the immunocytochemical staining between the primary tumor and distant metastases. This was explained by the significantly higher expression of PD-L1 in distant metastatic foci than that in the primary tumor tissue and indicated that the primary tumor was not adequate for determining the expression of PD-L1 in the metastatic areas 94. Real-time evaluation of the PD-L1 expression through liquid biopsy using immunocytochemical staining methods is a promising strategy that can potentially capture the dynamic nature of this biomarker compared with its evaluation in primary tumor tissue or metastatic foci.

Mazel *et al.* 12 examined the frequency of PD-L1 expression in patients with triple-negative and HER2-positive BC. CTCs were detected using the FDA-approved CellSearch® system followed by immunocytochemical staining of the enriched material with a ready-made cocktail of antibodies from CellSearch® and analysis on the Celltracks II® platform. PD-L1 expressing CTCs were detected in 11/16 patients with BC (68.8%). According to the authors, this specific subset was identified as metastatic cells with a high potential to avoid T-cell mediated lysis - a potent target for immunotherapy. The proportion of PD-L1-positive CTCs varied from 0.2 to 100% in individual patients. In line with this finding, the results of other studies 95 have highlighted the percentage of the PD-L1-positive CTCs to be in the range from 0 to 100%. Thus, these studies demonstrated that PD-L1 was often expressed on circulating metastatic cells in triple-negative and HER2-positive BC patients, in which patients with a high percentage of PD-L1-positive CTCs had to be identified as potential candidates for anti-PD-L1 therapy.

In a study by Schott *et al.* 95, the authors used real-time liquid biopsy to determine PD-L1 and PD-L2 expression in the CTC of 72 patients with BC using the maintrac® method. The average amount of CTC was 55/100 μL of blood (in the range from 5 to 805), CTC expressing PD-L1 were found in 94.5% of patients with BC; in patients expressing PD-L1 and PD-L2, the cell fraction of the PD-L1-positive CTCs was significantly higher than the fraction of the PD-L2-positive CTCs (54.6% versus 28.7%; p <0.001). In contrast with the study by Mazel *et al.*, Schott *et al.* were able to detect PD-L1-positive CTCs in patients without metastases, which might allow decisions to be already made when adjuvant therapy is prescribed. Moreover, patients with non-metastatic breast cancer had significantly more PD-L1-positive CTCs than patients without metastasis (median 75% versus 61.1%; p <0.05). The number of CTCs did not correlate with the other clinical and pathological parameters (age, size of the primary tumor, the presence of metastases in the lymph nodes, molecular subtype, predisposition. The authors performed a dynamic characterization of the PD-L1 expression on CTC and sequential monitoring of the treatment response in a patient with primary breast cancer. CTC numbers, as a signature of the successful immunotherapy, were decreased, and a percentage of the PD-L1-positive CTC was significantly reduced. After discontinuing ICI, the percentage of the PD-L1-positive CTC's continuously increased and a recurrence disease state was noted.

Thus, testing of PD-L1 in breast cancer tissue currently lacks standardization to encompass the diversity in the assays (IHC, gene expression), antibodies for testing, assessment systems and thresholds for classifying the PD-L1 expression (positive or negative), tumor microenvironment compartments included analysis (tumor cells, immune cells, or both) and nature of the tumor samples (primary, metastatic), along with a lack of appropriate widespread antibody platforms for testing PD-L1. Besides, there is currently no data on the effect of pre-analytic variables, including fixation time, type of fixative and storage on the reproducibility of testing PD-L1 in BC. The ratio between the expression of PD-L1 on CTC and the corresponding tumor tissue remains to be explored. An existing body of evidence suggests that the serum level of PD-L1 is directly related to the magnitude of the tumor burden 96 and the clinical outcome 97. Thus, a liquid biopsy holds promise as a feasible strategy for dynamic assessment and sequential monitoring of the PD-L1 expression in patients with BC, avoiding the disadvantages of a solid tumor biopsy and potentially providing a real-time picture of PD-L1. Given the small number of studies, further experiments are necessary, particularly determining the relationship between the expression of PD-L1 by CTC and tumor tissue and in-depth dynamic characterization of the expression of PD-L1 CTC of BC during immunotherapy.

## Lung cancer

In the treatment of non-small cell lung cancer (NSCLC), immunotherapy takes an ever-stronger position from year to year. Starting with use nivolumab (PD-L1 all corner, second line therapy, CheckMate-017, CheckMate-057 98), pembrolizumab (PD-L1 tumor proportion score ≥1%, second and further lines, KEYNOTE-010 99) and atezolizumab (PD-L1 all corner, second and further lines, OAK 100) after first line therapy with breakthrough results in increasing 5-year OS 101, there are currently a large number of immune-oncological first line treatment opportunities for both squamosus and not-squamosus mNSCLC. Pembrolizumab (KEYNOTE-024 102), atezolizumab (IMpower-110 103), or cemiplimab (EMPOWER-1 104) for PD-L1+ treatment-native patients can be used in monotherapy with significantly better tolerance and fewer adverse events. A recently published CITYSCAPE trial 105 has shown the efficacy of combination atezolizumab and tiragolumab for patients with hyper PD-L1 (≥50%) expression versus atezolizumab monotherapy with placebo. The use of immunotherapy in combination with standard platinum and/or pemetrexed chemotherapy has shown an increase in overall survival with better results at a PD-L1 expression level ≥50% (KEYNOTE-189 106, KEYNOTE-407 107, IMpower-150 108, IMpower-132 109) as well as the use of combinations of immuno-oncological drugs (СheckMate-227 110). Otherwise, at the same time, the use of a double combination of pembrolizumab + ipilimumab did not increase the median OS and PFS and was associated with a much higher incidence of grade 3-5 adverse events (KEYNOTE-598 111). Ongoing clinical trials on the use of immunotherapy in combination with chemoradiation therapy for patients with locally advanced NSCLC (IIIA-C stage, PACIFIC trial 112, KEYNOTE-799 113). Thus, the determination of PD-L1 expression in NSCLC directly affects the choice of therapy for the first and subsequent lines, the duration and quality of life of patients.

Lung cancer is one of the most common malignant neoplasms (12.9% of all new malignancies in the world) 114. Late diagnosis occurs in 70-80% of cases and is associated with a low patient survival rate 115. Quantifying and profiling CTCs are especially important for lung cancer patients. The relationship between the existence of CTCs in the peripheral blood of lung cancer patients and a poor prognosis has been demonstrated in most studies, regardless of the method of CTC isolation. The presence of CTC appears to correlate with the TNM stage 6, 116-118*,* lower disease-free and overall survival 119, 120. These findings have been confirmed by meta-analyses involving a large number of patients 121, 122.

It is necessary to take into account that the detection of CTCs in the case of non-small cell lung cancer is difficult owing to their small number and the presence of non-epithelial characteristics 123. A decrease in the number of CTCs was associated with a good response to cytostatic treatment and a longer overall and relapse-free survival 121, 122. Additionally, the dynamics of a number of CTCs in the course of radiation therapy 124 and targeted therapy (gefitinib and erlotinib) 125, 126 also correlated with a tumor. With an increasing number of possible treatment regimens, including target therapy and immunotherapy, determination of the molecular profile of NSCLC is critical at every stage of the disease progression because it is necessary to identify biological changes that cause drug resistance and affect treatment decisions. To date, two anti-PD-1 (nivolumab and pembrolizumab) antibodies and one anti-PD-L1 antibody (atesolizumab) are registered by the FDA as drugs for the treatment of NSCLC 127, 128. There are ongoing clinical trials on the use of these drugs in the first, second and third line of therapy in patients with NSCLC 129, 130. Previous studies convincingly demonstrated that not all patients with advanced and disseminated NSCLC benefit from treatment with these drugs. Only 20% of the entire cohort of patients responded to the use of ICI, which emphasized a need for the correct selection of patients for this expensive therapy 131.

Clinically, the isolation and identification of PD-L1-positive CTCs have been introduced as a challenging issue in the case of NSCLC because neutrophils and other immature myeloid subsets have a low to absent expression of CD45 and also express PD-L1, which make it challenging to identify the CTCs and leads to potentially false-positive results 132. Recently, PD-1 and PD-L1 expression across various subtypes of lung cancer cells, including squamous cell carcinoma, adenocarcinoma, and large cell carcinoma, were investigated 133. In this study, a predictive value of PD-L1 and PD-1 expression by CTC was highlighted in patients with metastatic lung cancer before treatment and after three chemotherapy cycles. The presence of PD-1-positive CTCs correlated with shorter PFS; however, because of the small number of patients, final conclusions regarding the prognostic value of the expression of this marker cannot be made.

Changes in the CTC number in patients with non-resectable NSCLC treated with chemo-radiation therapy regimens have been reported in various studies. The presence of CTCs was detected in 100% of samples post-irradiation in comparison with that of 93% at the pre-treatment stage 134. It was also found that an increased expression of PD-L1 by CTCs was associated with a poor prognosis. The results were consistent with those of Wang *et al.* 135, who reported on the effect of radiation therapy on the expression of PD-L1. In is study, whole blood from the non-metastatic NSCLC patients was collected before, during, and after the radiation or chemoradiation and processed using a microfluidic chip. PD-L1 expression in CTCs was assessed by immunofluorescence and qPCR and monitored through the course of treatment. PD-L1-positive CTCs were detected in 25 out of 38 samples (69.4%). After initiation of the radiation therapy, the proportion of PD-L1-positive CTCs significantly increased, indicating an up-regulation of PD-L1 in tumor cells in response to the radiation. Gene expression analysis revealed that the higher levels of PD-L1 were associated with poor prognosis. The authors concluded that CTCs can be used to monitor dynamic changes of PD-L1 during radiation therapy, which can potentially inform on the prognosis of a response to treatment.

Ilié *et al.* 136 reported on the enrichment of CTCs and circulating white blood cells (WBCs) from peripheral blood samples from 106 NSCLC patients. The PD-L1 status of tumor tissue and CTCs, tumor-infiltrating WBC, and WBC of peripheral blood was studied, and their high interrelation was shown. A trend towards worse survival in patients receiving first-line cisplatin-based chemotherapy treatments, whose tumors expressed PD-L1 in CTCs or immune cells was found, similar to the effects of PD-L1 expression in matched-patient tumors. At the same time, in the study by Janning *et al.* 137, in contrast to the results reported by Ilié *et al.* 136, the percentage of the PD-L1-positive CTCs did not correlate with the percentage of PD-L1-positive in the biopsies determined by IHC. Also, the data demonstrated considerable heterogeneity in the PD-L1 status of CTCs in NSCLC patients.

Nicolazzo *et al.* 138 enrolled 24 patients at stage IV of NSCLC treated ICI Nivolumab. At a baseline and three months of treatment, the presence of CTCs and the expression of PD-L1 on their surface were found, and this was associated with poor treatment outcomes. Conversely, although CTCs were found in all patients six months after the treatment, at this time, patients could be dichotomized into two groups based on the PD-L1 expression of CTCs. All patients with PD-L1-negative CTCs had a clinical benefit, whereas all patients with PD-L1-positive CTCs had progressive disease. This suggested that the persistence of PD-L1-positive CTCs might mirror a mechanism of therapy resistance.

Guibert *et al.* 139 prospectively collected blood samples from patients with advanced NSCLC before nivolumab treatment and at the time of progression. CTCs were isolated using a cell size-based technology, and PD-L1 expression was assessed by immunofluorescence on CTCs and immunohistochemistry on tissue biopsies. CTCs were more often found to be PD-L1-positive than tissue (83% versus 41%), and no correlation was observed between tissue and CTC PD-L1 expression. A high CTC number before treatment was associated with an increased risk of death and progression. The presence of PD-L1-positive CTC pretreatment did not significantly correlate with the outcomes, but a higher baseline PD-L1-positive CTC number (≥1%) was observed in the “non-responders” group (PFS < 6 months), and PD-L1-positive CTCs were seen in all patients at progression.

In a study by Kulasinghe *et al.*, CTCs were detected in 51.5% of blood samples of NSCLC patients, 64.7% of which were PD-L1-positive 140. In the advanced stage NSCLC patient cohort, PFS was not found to be associated with CTCs before therapy, nor the presence of PD‐L1 expression. The possible predictive value of PD-L1-positive CTCs was also studied in patients receiving treatment with the ICIs pembrolizumab, nivolumab and avelumab 141. In this study, a blood enrichment system based on different sizes of normal and tumor cells was used (Vortex HT chip). Because of the small number of patients, the study could not conclude the prognostic value of PD-L1 expression by the CTCs. Furthermore, Raimondi *et al.* 142 demonstrated that PD-L1-positive CTCs isolated from NSCLC patients were characterized by partial epithelial-mesenchymal transition phenotype and hypothesized that co-expression of PD-L1 and epithelial-mesenchymal transition cellular markers might represent a possible molecular background for the immune escape.

Analyzing a possible predictive and prognostic role of the expression of PD-L1/PD-1 by CTCs in patients with lung cancer, Raimondi *et al.* 143 noted the need to standardize the presentation of both clinical data and data regarding the blood processing (including the sampling, blood stabilization, storage time and temperature). Another problem pointed out by the authors of the review was the need to standardize methods for isolation of CTCs from samples of peripheral blood and their subsequent identification. Particularly, the most commonly used EpCAM-based CellSearch® System is designed to, first of all, identify cells of epithelial origin rather than CTCs, and therefore data collected with this system should be interpreted with caution. In addition, uniform approaches for the selection of antibodies utilized for the PD-L1 immunostaining are deemed necessary.

## Head and neck cancer

Head and neck squamous cell carcinoma (HNSCC) is traditionally associated with a high risk of recurrence and poor prognosis despite aggressive multimodal treatment strategies in clinical practice. Surgical methods, or radiation therapy, or chemotherapy, do not significantly increase patients' 5-year overall survival with a primary tumor, which remains at 40-50% 144. Moreover, there is a lack of treatment options for patients with metastatic or recurrent disease; the median OS after the diagnosis is less than one year. Platinum-based chemotherapy and monoclonal antibody cetuximab targeting epidermal growth factor receptor are common choices for recurrent/metastatic HNSCC 145. Nevertheless, the problem of treatment-related side effects has not been solved, and there is a significant amount of unmet need for improving treatment efficacy without further exacerbating toxicity.

The emergence of the human papillomavirus (HPV)-associated subset of HNSCC coupled with the rapid development of immunotherapy has motivated novel, immune-based approaches to treatment. It was shown that HNSCC is an immunosuppressive disease, and patients have lower absolute lymphocyte counts than healthy subjects 146, impaired natural killers cell activity 147, 148, and poor antigen-presenting function 149. Suppressive regulatory T cells secrete suppressive cytokines such as TGF-E and IL-10, express CTLA-4 and correlate with a poor prognosis, whereas the impairment of tumor-infiltrating lymphocytes (TILs) has a strong impact on clinical outcome, particularly in the case of HNSCC. Furthermore, HNSCC has a relatively high TMB 150, which is presumably owing to the production of altered and consequently antigenic proteins from mutated DNA, and can be predictive of the efficacy of ICIs 151.

The PD-1/PD-L1 pathway is a key mechanism of immune escape by tumor cells, so anti-PD1/PD-L1 agents boost the anti-tumor immune response and block the immunosuppressive signaling of tumors 152. The biological rationale for targeting the anti-PD1/PD-L1 pathway in HNSCC has been reinforced by recent large clinical trials, demonstrating improved outcomes from ICIs compared with standard of care therapy. KEYNOTE-012 was a phase Ib trial that was the first to demonstrate durable responses to pembrolizumab in patients with platinum-refractory R/M HNSCC with ≥1% PD-L1 expression with an overall response rate of 16% 153, 154. Soon after, data from the randomized phase III CheckMate 141 trial of 361 patients showed improved OS and quality of life relative to investigator′s choice of the standard of care systemic therapy for the platinum-refractory disease regardless of HPV status, presence or no prior cetuximab therapy and PD-L1 expression 155. The pembrolizumab activity in the platinum-refractory R/M HNSCC setting was subsequently confirmed in the Phase II KEYNOTE-055 156 and Phase III KEYNOTE-040 trials 157. In Phase III KEYNOTE-048 for the first-line recurrent/metastatic HNSCC, pembrolizumab significantly improved OS over EXTREME clinical trial with cetuximab. It is clear that such agents should be used to manage all patients who do not have contraindications. In this regard, the urgency of developing a correct method for assessing PD-L1 tumor status is increased.

In a study by Kulasinghe *et al.*, where blood samples from 23 patients with head and neck cancer (HNC) were examined for CTCs, CTC clusters were identified in 11/23 HNC samples. Notably, three of these patients had only CTC clusters and seven patients had both single and cluster CTCs. PD-L1 was found to be expressed (at least 1 CTC staining by immunofluorescence positive) in 6/11 (54.4%) samples. In two cases of HNC, all CTCs were PD-L1-positive. There was a significant difference in PFS between CTC-positive patients compared with the absence of CTCs in the investigated HNC cohort 140. In another study by Kulasinghe *et al.* 158, CTCs clusters expressing PD-L1 were found in a patient suffering from supraglottic squamous cell carcinoma. A previously developed technique 5, 12 utilizing spiral microfluidic technology 159 was used in this study. The detected clusters exhibited a medium-high expression of PD-L1 compared with a panel of known HNC cell lines 158.

Many scientific groups have confirmed the importance of using molecular analysis for molecular characterization of CTCs 13, 160-162. Real-time molecular analysis based on real-time PCR performed in a nucleic acid material (RNA or genomic DNA) isolated from the EpCAM-positive CTC fraction has been used to provide valuable information about the molecular characterization of CTC during gene expression 34, DNA methylation 35, 38 and the level of the DNA mutation 39. In a study by Strati *et al.* 13, RT-qPCR analysis was applied for the analysis of PD-L1 mRNA transcripts to evaluate the increased expression of PD-L1 in the EpCAM-positive CTCs fraction. The authors showed that the detection of CTCs with the expression of PD-L1 at the completion of the final treatment in HNSCC patients correlated with a higher risk of progression and mortality compared with the negative analogs of PD-L1.

## Gastrointestinal cancers

According to global cancer statistic reports, gastrointestinal malignancies, an important component of solid tumors, have a heavier cancer-associated burden 163. Currently, metastasis is the leading cause of malignancy-related deaths in gastrointestinal cancers 164. Even at the early stage, a considerable cohort of patients that undergo resection treatment develop a metastatic disease within five years post-surgery 165. This evidence points to a dormant metastatic process concurrent with the primary tumor genesis 166. Additionally, tumor cells with metastatic potential are shed from the primary tumor site during surgery and are circulated in the system, resulting in distant metastasis 167, 168.

Speaking of immunotherapy, one should not forget that its role in the treatment of gastrointestinal tumors remains controversial despite a certain number of clinical studies. For PD-L1-positive esophageal and esophagogastric junction cancers, ICIs have shown their efficiency not only in second and subsequent lines of therapy (KEYNOTE-061 169, KEYNOTE-059 170, KEYNOTE-181 171), but recently in the first line in combination with chemotherapy. Thus, in KEYNOTE-590 study 172, pembrolizumab combined with chemotherapy provided superior OS, PFS, and ORR with a manageable safety profile in patients with untreated, advanced esophageal and esophagogastric junction cancer with hyper PD-L1 expression. On the contrary, in gastric cancer (GC), the use of pembrolizumab in previously untreated PD-L1^+^ patients, both in monotherapy and in combination with chemotherapy, did not give any statistically significant benefit (KEYNOTE-062 173) and was effective only for patients with MSI-tumors. Adding nivolumab to standard first line therapy (XELOX\FOLFOX) in patients with HER2-negative GC with has led to significantly improved progression-free and overall survival over chemotherapy alone (CheckMate-649 174), whereas the use of similar therapy regimens without prior selection of patients according to the level of PD-L1 expression produced negative results in the ATTRACTION-4 175 trial. For the case of hepatocellular carcinoma (HCC), there is a modest number of clinical studies, which, however, have shown the effectiveness of the use of immunotherapy in first line of therapy (atezolizumab + bevacizumab, IMBrave150 176) and in subsequent lines in combinations (ipilimumab + nivolumab/pembrolizumab, Abstract 330 177). For pancreatic and colorectal cancers, there is still no optimal cohort of patients for immunotherapy, as well as for PD-L1 expression testing.

The predictive value of PD-L1 IHC is still under debate because some patients with PD-L1 negative status tumors of various types of cancer, including GC, respond to anti-PD-1 therapy 178, 179. In line with these observations, Cheng *et al.* 180 found that in GC patients with PD-L1-negative tumor tissue, the imaging flow cytometry signal analysis of isolated CTC was as varied as that of IHC staining, suggesting that the expression of CTC PD-L1 was useful in the immunophenotypic differential diagnosis of tumors and could be a potential candidate for anti-PD-1/PD-L1 immune checkpoint therapy 143.

Because PD-L1-positive CTCs have been recognized as a biomarker for therapeutic efficacy of ICI in NSCLC, it is hypothesized that CTCs can also be a predictive marker in GC immune checkpoint therapy 181. It was identified that after checkpoint blockade therapy, CTC enumeration can be a useful tool to predict poor prognosis in advanced GC patients 132. In line with this, Yue *et al.* 182 demonstrated that the PD-L1 level in CTCs could be a potential predictor for PD-1/PD-L1 blockade therapies in patients with advanced gastrointestinal tumors. The disease control rate in the patients with PD-L1-high CTC number was much higher than those with a lower level. Additionally, several PD-L1-high CTCs at the baseline had predictive values for PFS. Moreover, the dynamic changes in the number of PD-L1-positive CTCs can reflect the real-time response status. However, in this study, the changes in the total number of isolated CTCs, PD-L1-positive CTC, and PD-L1-high CTC significantly correlated with the clinical outcomes. This cohort study highlighted the advantage of using CTC-PD-L1 over the tumor PD-L1, which can provide baseline information for treatment response prediction in a time-efficient manner.

Similarly, Cheng and colleagues isolated CTCs from 32 GC patients and demonstrated that a total CTC pool and CTC-PD-L1 were highly correlated with the clinical outcome of checkpoint blockade therapy. Additionally, a high number of PD-L1-positive CTCs in advanced GC patients was predictive of better five-year OS 180. Taken together, the clinical evidence supports a potential application of PD-L1 expressing CTCs as a reliable prognostic biomarker for checkpoint blockade therapy in GC. Moreover, recent advances in single-cell molecular profiling of CTCs and tumor cells using next-generation sequencing may also provide a pool of tools to validate predictive biomarkers, including CTC-PD-L1.

In pancreatic ductal adenocarcinoma, an association between the presence of PD-L1 expression on isolated CTCs and patient outcome was described in few studies. The numeration and PD-L1 expression levels of CTC were analyzed in a clinical study involving 35 patients at various advanced stages of gastrointestinal tumors including pancreatic ductal adenocarcinoma: 74% (26/35) of patients had PD-L1-positive CTC and 60% (21/35) had at least one PD-L1-high CTCs 182. Comparing the CTC number and expression of PD-L1 on isolated CTC before and after treatment with a PD-L1 inhibitor, patients with a stable-disease status showed a significant reduction in the number of CTC (25 versus 15) and PD-L1-high CTC (40% versus 6.67% of PD-L1 expression) in comparison with patients with progressive disease status. The results suggested that the abundance of PD-L1-high CTCs at a baseline might serve as a predictor to screen patients for PD-1/PD-L1 blockade therapies and measure the dynamic changes of CTC to indicate the therapeutic response of the initial cycle of treatment.

Recently, evaluation of the expression of PD-L1 on captured CTCs in patients with HCC opened a new way to manage patients undergoing immunotherapy. In a prospective cohort of 92 patients (8 healthy control, 11 non-malignant liver disease, 73 HCC), the enumeration and characterization of the phenotype of captured CTCs were analyzed 183. Considering the expression of PD-L1 on isolated CTCs, PD-L1^+^ CTCs classified HCC patients at early-stage (3/39 with PD-L1^+^ CTCs) and advanced/metastatic disease (23/34 with PD-L1^+^ CTCs) with sensitivity and specificity of 67.7% and 92.3%, respectively. Additionally, prior to the immunotherapy, 3 of 6 patients receiving anti-PD-1 therapy that successfully responded to therapy showed PD-L1-positive CTCs compared with only 1 of 3 non-responders.

Based on this evidence, in a larger cohort of 87 patients with various stages of HCC (49 early‐stage, 22 locally advanced, and 16 metastatic), PD-L1^+^ CTCs were identified in 4 of 49 early‐stage patients, 12 of 22 (54.5%) locally advanced and 15 of 16 (93.8%) metastatic patients. Moreover, the analysis of the PD-L1 expression on CTCs could be used to accurately discriminate between early-stage and locally advanced/metastatic HCC (sensitivity at 71.1%, specificity at 91.8%) 184. In 10 patients who received PD‐1 inhibitor, the treatment-responsive patients (n = 5) demonstrated PD-L1^+^ CTCs at a baseline compared with only 1 of 5 non-responders with a progressed disease within four months of starting treatment. This evidence indicates a robust positive association between PD-L1^+^ CTCs and positive treatment response in patients with HCC that received anti‐PD‐1 inhibitor.

## Melanoma

In melanoma, immunotherapy with ICIs has shown benefit in a subset of melanoma patients 185, 186. Whilst targeted treatment such as BRAF and MEK inhibitors have shown transient responses, ICI therapies have led to long-lasting and durable responses. However, there remains a need for predictive biomarkers of response to ICI therapy. Several biomarkers have been introduced for guiding patient selection for immunotherapy, such as PD-L1 expression and TMB.

Thus, the use of pembrolizumab as a first line therapy significantly increased ORR and PFS in patients with expression PD-L1 rather than in its absence (KEYNOTE-001 187). The benefit from the use of nivolumab alone or in combination with ipilimumab (CheckMate 066 188, CheckMate 067 189) was also higher in groups of patients with PD-L1 expression level ≥5%.

One of the challenges is the enrichment and isolation of CTCs and comprehensive characterization of CTCs for biomarkers of response to therapy. This challenge has been met by the recent advances in microfluidic technology. Aya-Bonilla *et al.* 190 demonstrated that melanoma CTCs could be efficiently isolated and characterized using spiral chip technology. They recently compared two microfluidic technologies: Parsotix (Angle PLC) and ClearCell (Biolidics, Singapore) to capture and characterize melanoma CTCs. This study highlighted the heterogeneity of CTCs and multiple, non-overlapping populations 191. Furthermore, facile detection of CTCs and the evaluation of PD-L1 expression has been reported in various studies 192.

CTCs have been used to quantify the response to therapy in melanoma. Hong *et al.* 193 analyzed a panel of 19-gene RNA signatures in enriched CTCs using microfluidic technologies, which were capable of monitoring the tumor burden in patients. In a study by Khattak *et al.* 194 using flow cytometry for the identification and characterization of CTCs based on melanoma markers (MCAM, MCSP) in metastatic melanoma, the authors found that PD-L1 expression buy CTCs was associated with a response to therapy and longer PFS. In a subsequent study by Khattak *et al.* 195, PD-L1-positive CTCs were identified in 64% of total CTCs and those patients had a significantly longer PFS. The survival at 12 months for patients with detectable PD-L1-positive CTCs was 76% compared with 22% for patients with PD-L1-negative CTCs. They also found that the expression of PD-L1 on CTCs was an independent predictive biomarker of PFS. In line with these findings, Yue *et al.* 182 applied a dynamic expression level of PD-L1 and demonstrated that the presence of PD-L1^+^ CTCs at a baseline could serve as a predictor for screening patients for anti-PD-1/PD-L1 therapy and measuring CTC expression levels probably indicated real-time changes to therapies.

These data suggest that the PD-L1 expression on CTCs could be used to predict the response to anti-PD-1/PD-L1 therapy (e.g., pembrolizumab) in melanoma. However, larger validation studies are needed to identify whether liquid biopsy has the potential to be used as a tool for identifying patients likely to respond to therapy. These investigations need to include a direct comparison of liquid biopsy and solid tumor biopsy for PD-L1 expression scoring and measurement against the response to anti-PD-1/PD-L1 therapy to determine the clinical utility of this assay.

The studies, discussed in the current review, where immunotherapy was combined with CTC assessment are summarized in Table 1.

## Summary and outlook

In this review, we discussed the role of immunotherapy in a number of solid malignancies and the need for the development of non-invasive liquid biopsy approaches for treatment management. Although for tumors of some localizations such as NSCLC, or TNBC, PD-L1 testing is really critical for using immuno-oncological drugs in first line treatment as mono-therapy or in combinations with the best efficacy and toxicity profile, for others such as melanoma, immunotherapy is effective in itself, is minimally associated with the level of PD-L1 expression and, apparently, does not require its dynamic assessment over time. The effectiveness of checkpoint inhibitors for other tumors such as colorectal and pancreatic cancer is still questionable and requires further research. Genomic subtypes of gastrointestinal tumors, in particular gastric cancer, differing in their genomic characteristics, create controversy in the response of such tumors to treatment with checkpoint inhibitors and cause mixed results in clinical trials. At the same time for head and neck cancers, it is important not to miss the optimal testing time to determine the better therapy for second and subsequent lines.

From a clinician's point of view, the main problem in testing CTCs is to determine the “ideal” cohort of patients for whom the time and financial costs of testing itself and subsequent immunotherapy will be commensurate with the increase in the effectiveness of the treatment. This is difficult until the indications for immunotherapy of solid tumors have not been finally determined and the localization of tumors for which such treatment is obviously ineffective is not completely ruled out. The clinical benefit of studying the expression of immune checkpoints on CTCs in real time is that it can probably predict the moment that resistance to therapy with checkpoint inhibitors is formed, but at the moment it is poorly understood and requires further discussion.

Immunotherapies have been hailed as a 'game-changer' in the field of cancer therapies. However, there remain some challenges in identifying which patients would best respond to ICB therapy. The challenge remains in assessing the dynamical genesis of a tumor using static, single timepoint tissue biopsy samples, because a repeat biopsy is often challenging to perform and carries associated risks for the patient. A solution for repeated measurements during the course of therapy could be the analysis of liquid biopsy samples of patients. The analysis of CTCs in the peripheral blood of patients represents such an approach, which has FDA approval for the enumeration of CTCs. Although the enumeration of CTCs has been associated with PFS and OS across a number of solid tumors, the characterization of these cells becomes increasingly more important. Recent reports have shown frequent expression of immune checkpoint proteins on CTCs such as PD-L1, which can be assessed prior to and during the course of therapy to understand dynamic tumor genesis in real time. While these data still emerge, clinical trials incorporating CTC enumeration, characterization and changes over the course of immunotherapy may be useful in determining whether CTCs can be used as a surrogate marker of response to therapy. Recent literature supports the notion that CTCs may provide an immune escape mechanism for tumors to evade the body's immune system and further investigation is required to determine the role of neutrophils and other immune cells which co-locate and cluster with CTCs. At the same time, standardisation of protocols for isolation/detection and analysis of CTCs is highly required.

## Figures and Tables

**Table 1:** Crucial findings on CTCs as biomarkers in cancer immunotherapy.

Type of cancer	Cancer form	Type of immunotherapy	Purpose of the study	CTC detection/isolation technique	CTC prognostic significance	Ref
Prostate cancer 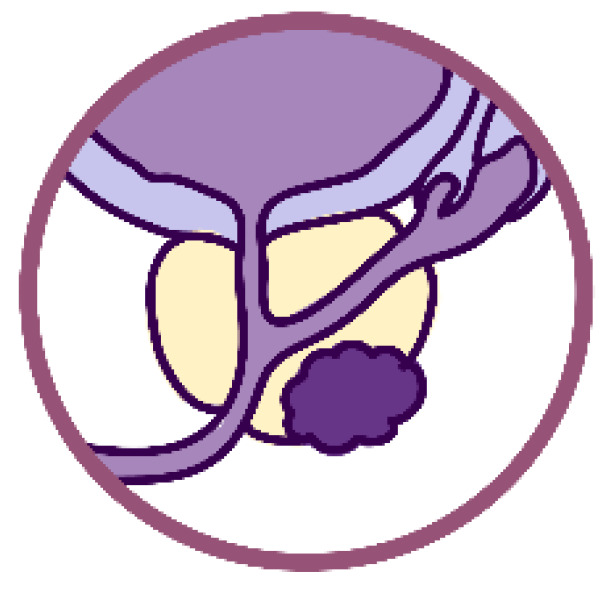	Castration-resistant, none-metastatic form	Combination of anti-tumor vaccination and pembrolizumab	To analyze changes in checkpoint receptor expression on antigen-specific CD8+ T-cells and the effect of PD-1 blockade on immune response, and to analyze PD-L1 expression on CTCs.	Flow cytometry	PD-L1 expression on CTCs increased after vaccination, which was associated with the development of T-cell immunity and longer PFS.	25
	Metastatic castration-resistant form	Nivolumab, ipilimumab	To investigate if tumors of patients with AR-V7+ CTCs had more DNA-repair deficiency mutations, and therefore were potentially more sensitive ICB therapy.	AdnaTest	Presence of AR-V7+ CTCs was associated with CTCs heterogeneity, which correlated with likelihood of favorable response to immune-checkpoint inhibition.	26
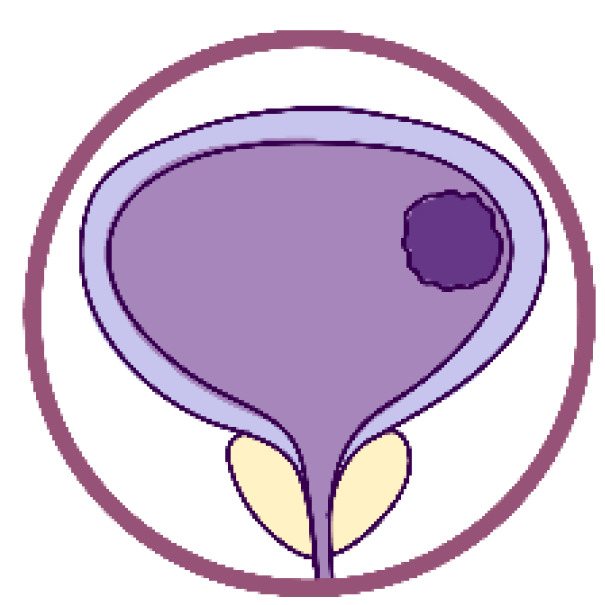 Urothelial bladder cancer	Metastatic and muscle invasive forms	MPDL3280A anti-PD-L1 monoclonal antibody	To examine the efficiency of atezolizumab for the treatment of patients with urothelial bladder cancer. A biomarker analysis was performed, which included the analysis of CTCs and TILs for the expression of PD-L1.	N/A	The amount of PD-L1^+^ TILs was associated with worse median OS. The 43% response rate was achieved in patients with PD-L1^+^ IHC.	29
Ovarian cancer 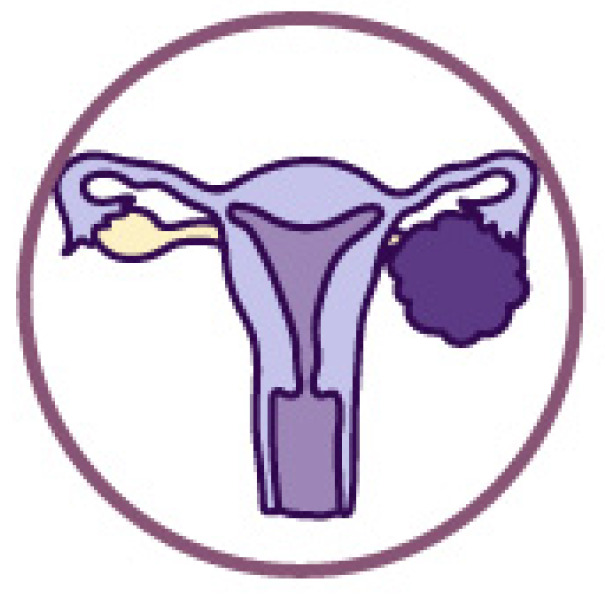	Metastatic form	HER2-targeted cancer vaccine	A phase I clinical trial in patients, which included assessment of CTCs as one of the major biomarkers of therapy response.	N/D	A decrease in the number of CTCs by 40%, 83% and 100% at 12, 28 and 48 weeks post-treatment was identified.	66
	Advanced platinum-resistant ovarian cancer	GL-ONC1 oncolytic immunotherapy	To identify clinical benefits of VOV monotherapy.	N/D	The absence of CTCs in peripheral blood directly correlated with the positive therapeutic effects of GL-ONC1 therapy.	67
Lung cancer (NSCLC) 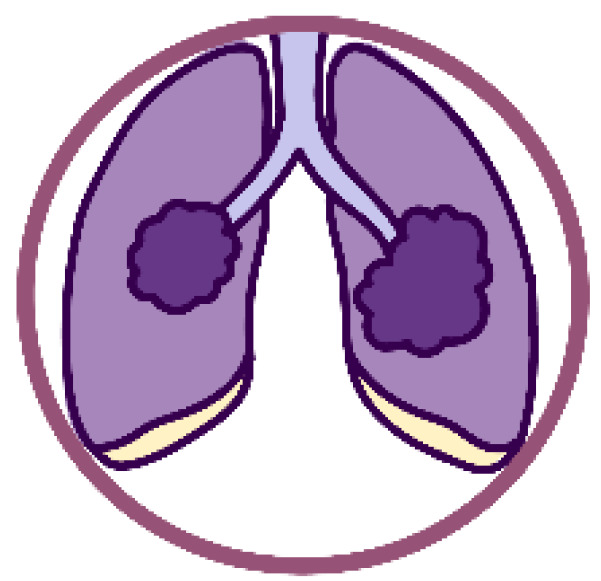	TNM stages IIB-IV, including both metastatic and none-metastatic patients	Pembrolizumab, nivolumab, atezolizumab	To test an epitope-independent method (Parsortix© system) and utilize it to assess PD-L1 expression of CTCs from NSCLC patients undergoing PD-1/PD-L1 inhibitors therapy.	Cellsearch system and Parsortix system	Upon disease progression, all patients demonstrated an increase in PD-L1^+^ CTCs, while no change or a decrease in PD-L1^+^ CTCs was observed in responding patients. An increase of PD-L1^+^ CTCs had a potential to predict resistance to PD-1/PD-L1 inhibitors.	137
	TNM stage IV, metastatic form	Nivolumab	To investigate if liquid biopsy might allow real-time sampling of patients for PD-L1 through the course of immunotherapy. PD-L1 expressing CTCs were assessed at baseline, at 3 and 6 months post-treatment.	Cellsearch system with anti-human B7-H1/PD-L1 conjugated antibody	A high CTC number was associated with poorer prognosis. At baseline and at 3 months of treatment, the presence of PD-L1^+^ CTCs was associated with poor patient outcome.	138
	Advanced metastatic form	Nivolumab	To investigate if liquid biopsy might allow real-time sampling of patients for PD-L1 through the course of immunotherapy.	ISET technology	Patients with high CTC count experienced worse outcomes. The presence of PD-L1^+^ CTCs had no significant impact on PFS. Patients with PD-L1^+^ CTCs at baseline were more often non-responders compared to PD-L1- patients.	139
	Metastatic form	Pembrolizumab treatment	To determine if PD-L1 expression on CTCs can serve as a predictive biomarker of clinical benefit and response to treatment with the PD-1/PD-L1 inhibitor pembrolizumab.	Ficoll-Paque Density Gradient Media	PD-L1^+^ CTCs were identified in 64% of total CTCs. Patients with PD-L1+ CTCs had significantly longer PFS.	195
Hepatocellular carcinoma 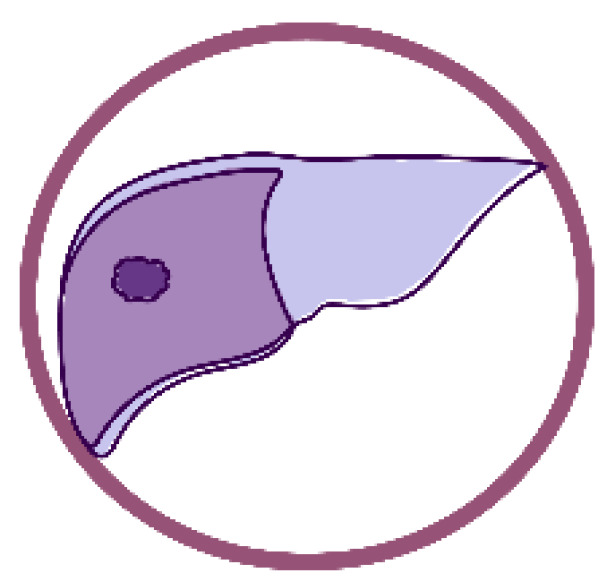	Early, locally advanced and metastatic stages	Anti-PD1 therapy (unspecified)	To detect PD-L1 expressing HCC CTCs, and investigate its role as a prognostic biomarker.	NanoVelcro Chip	The study indicated a strong positive association between the presence of PD‐L1+ CTCs and positive treatment response. Analysis of PD-L1 expression on CTCs discriminated early stage from locally advanced/metastatic.	183
Different gastrointestinal tumors 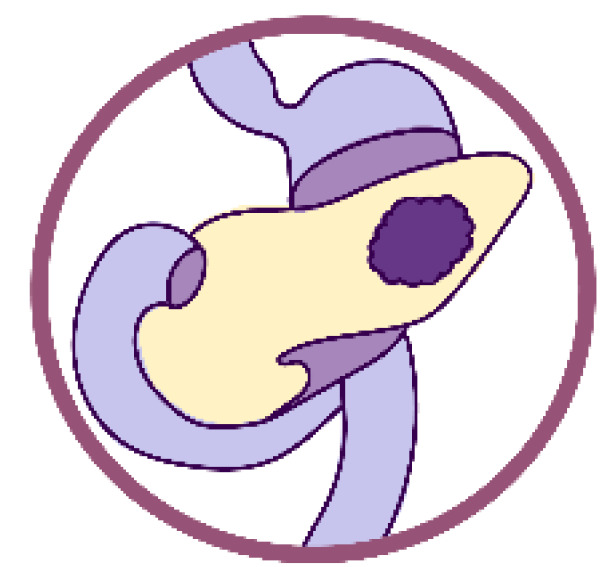	Metastatic form	IBI308 PD-1 monoclonal antibody therapy	To determine if PD-L1 expression on CTCs could serve as an alternative biomarker of tumor response for predicting outcomes and monitoring PD-1 blockade therapies.	The Pep@MNPs isolation system	Before treatment: 74% of patinets had PD-L1^+^ CTCs, 60% had at least 1 high-positive PD-L1 CTC. After treatment: significant reduction of PD-L1^+^ CTCs and high-positive PD-L1 CTCs (40% *vs.* 6.67%). CTCs might be used as a baseline predictor to screen patients for PD-1/PD-L1 blockade therapies and monitor therapeutic response.	182

**Abbreviations:** AR-V7: androgen receptor splice variant 7; AR-V7^+^: androgen receptor splice variant 7 positive; B7-H1: B7 homolog 1; CD8^+^: cluster of differentiation 8 positive; CTC: circulating tumor cell; DNA: deoxyribonucleic acid; HCC: hepatocellular carcinoma; HER2: human epidermal growth factor receptor 2; ICB: immune checkpoint blockade; IHC: immunohistochemistry; NSCLC: non-small cell lung cancer; PD-1: programmed cell death protein 1; PD-L1: programmed death-ligand 1; PFS: progression-free survival; TIL: tumor-infiltrating lymphocyte; TNM: tumor, nodes and metastases; VOV: vaccinia oncolytic virus

## Abbreviations

AR-V7: androgen receptor splice variant 7
AR-V7^+^: androgen receptor splice variant 7 positive
B7-H1: B7 homolog 1
CD8^+^: cluster of differentiation 8 positive
CTC: circulating tumor cell
DNA: deoxyribonucleic acid
HCC: hepatocellular carcinoma
HER2: human epidermal growth factor receptor 2
ICB: immune checkpoint blockade
IHC: immunohistochemistry
NSCLC: non-small cell lung cancer
PD-1: programmed cell death protein 1
PD-L1: programmed death-ligand 1
PFS: progression-free survival
TIL: tumor-infiltrating lymphocyte
TNM: tumor, nodes and metastases
VOV: vaccinia oncolytic virus
